# Critical Temperatures for Vibrations and Buckling of Magneto-Electro-Elastic Nonlocal Strain Gradient Plates

**DOI:** 10.3390/nano11010087

**Published:** 2021-01-03

**Authors:** Giovanni Tocci Monaco, Nicholas Fantuzzi, Francesco Fabbrocino, Raimondo Luciano

**Affiliations:** 1 Department of Civil, Chemical, Environmental and Materials Engineering, University of Bologna, 40136 Bologna, Italy; giovanni.toccimonaco@studio.unibo.it; 2 Engineering Department, Parthenope University, 80133 Naples, Italy; raimondo.luciano@uniparthenope.it; 3 Department of Engineering, Telematic University Pegaso, 80132 Naples, Italy; francesco.fabbrocino@unipegaso.it

**Keywords:** smart nano-plates, semi-analytical solution, critical temperatures, buckling, vibrations

## Abstract

An analytical method is presented in this work for the linear vibrations and buckling of nano-plates in a hygro-thermal environment. Nonlinear von Kármán terms are included in the plate kinematics in order to consider the instability phenomena. Strain gradient nonlocal theory is considered for its simplicity and applicability with respect to other nonlocal formulations which require more parameters in their analysis. Present nano-plates have a coupled magneto-electro-elastic constitutive equation in a hygro-thermal environment. Nano-scale effects on the vibrations and buckling behavior of magneto-electro-elastic plates is presented and hygro-thermal load outcomes are considered as well. In addition, critical temperatures for vibrations and buckling problems are analyzed and given for several nano-plate configurations.

## 1. Introduction

In recent years research has focused heavily on MEMS (Micro-Electro-Mechanical-System) and NEMS (Nano-Electro-Mechanical-System). This interest is mainly due to the wide variety of applications in which these devices could be used [1,2,3,4]. These structures, such as nanoplates, nanorods, and nanobeams [5], can be used in medicine [6], electronics [7], aerospace [8] and in civil construction [9], where linear and nonlinear theories are generally needed [10]. The behavior of this type of structures cannot be well described through the classical theories of continuous mechanics, as they are based on the principle of location of stresses. Due to the size of these devices, the effects induced by nanoscales must be taken into account [11,12]. Then to improve the ability of new devices and systems made with these smart materials, it is necessary to accurately investigate the mechanical behavior of these advanced structures [13,14]. Non-local theories have been widely used for the study of nanostructures since Eringen developed his theory of non-local elasticity [15]. These theories consider the nano-scale effects thanks to the introduction of one or more length scale parameters in addition to the well known linear elastic Lamé parameters [16,17,18,19]. The classification of nonlocal theories is generally presented as: strain gradient [20,21,22,23], stress gradient [24], modified strain gradient [25,26,27], couple stress [28], modified couple stress [29,30], integral type [31,32] and micropolar [33,34,35]. Article [36] offers a overview on unified continuous/reduced-order modeling and non-linear dynamic theories for thermomechanical plates. Kim in [37] developed a matrix method for evaluating effective elastic constants of generally anisotropic multilayer composites with various coupled physical effects including piezoelectricity, piezomagnetism and thermoelasticity. In [38,39,40] a nonlocal nonlinear first-order shear theory is used for investigating the buckling and free vibration of magneto-electro-thermo elastic (METE) nanoplates under magneto-electro-thermo-mechanical loadings. Mota in [41] investigated the influence of shear factor used in the context of the first-order shear deformation theory on functionally graded porous materials. In [42] free and forced vibration of a functionally graded piezoelectric plate with the properties of the material varying along the thickness are investigated. Combined asymptotic-tolerance modelling of dynamic and stability problems for functionally graded shells was given in [43,44]. In [45] are studied the pyroelectric and pyromagnetic effects on multiphase MEE cylindrical shells subjected to a uniform axisymmetric temperature using semi-analytical finite element procedures. Eremeyev et al. in [46,47] investigate the effect of flexoelectricity and flexomagnetic on nanobeams. Using FEM formulation [48,49] analyzed free vibration of orthotropic cross-ply nanoplates and nanowires. Ebrahimi in [50] studied buckling behaviour of magneto-electro-elastic functionally graded nanobeams using higer-order beam theory and Eringen’s non-local elasticity. Also in [51] the behaviour of MEE nanobeams is investigated using Eulero-Bernoulli beam theory and including surface effects. In [52] the focus is on free vibration of laminated circular piezoelectric plates and discs using the weak form of the equations of motion. In [53] using the third order shear deformation plate theory, the bending, buckling, free and forced vibration behavior of a nonlocal composite microplate is analyzed. Functionally graded microplates using Kirchhoff plate’s theory and straing gradient theory with only one length scale parameter are studied in [54]. In [55] theory of elasticity including surface stresses is used to study behaviour of shells with nano-scaled thickness. In [56] Kirchhoff plate’s theory and the modified flexoelectric theory are used to study the nonlinear free vibration of Functionally Graded (FG) flexoelectric nanoplate by taking into account size-dependent effects. In [57] a finite element model based on a higher-order plate’s theory is developed to study static and free vibration problem of magneto-electro-elastic plates. In [58] the dynamic problem of thin elastic plates resting on elastic foundation is studied using tolerance averaging method. Vibrations on periodical structures as well as bad gaps problems in dynamics are still an open topic thoroughly discussed by several researchers [59,60,61,62]. In [63] the flexural vibration band gaps in periodic beams is investigated using differential quadrature method, moreover the influence of shear deformation on the gaps is analyzed. Similarly, natural frequencies of structures made of period cells was presented for beams in [64,65] and for plates in [66,67]. In [68] the aim is the problem of vibration of band gaps in periodic Mindlin plates and it is solved using spectral element method. Also in [69] is studied the problem of vibration of band gaps in periodic plates, but is solved using differential quadrature element method. Vibration band gap of stiffened thin plate is studied also in [70] and is solved using center finite difference method. In [71,72] the problem of flexural wave propagation of a periodic beam is investigated.

The aim of this paper is the study of buckling and free vibrations of functionally graded nano-plates in hygro-thermal environment. For buckling analysis it will investigate the influence of external applied electric and magnetic potentials on critical load that leads to the instability of the plate, while in the case of dynamic analysis will be studied the behavior of natural vibration frequencies and how they are influenced by external potentials and temperature. Through the graphs will also identify the critical temperature, which corresponds to the temperature at which the vibration frequencies become zero. This paper is structured as described below. After the introduction section, the theoretical background for functionally graded (FG) thin plates in hygro-thermal environment is developed introducing also the non-linear terms of von Kármán that allow to perform the linear analysis of buckling. Using second order strain gradient theory non local effect are take into account. The following is a small paragraph showing how the electrical and magnetic potentials are approximated. Using Hamilton’s principle, for the case of METE (magneto-electro-thermo-elastic) materials, the equations of motion are obtained. The analytical solution is obtained using Navier developments in double trigonometric series. Then the results obtained through calculation code implemented in MATLAB for buckling and free vibration are provided. Finally, a conclusion section is reported at the end of this paper.

## 2. Theoretical Background

**Figure 1:**
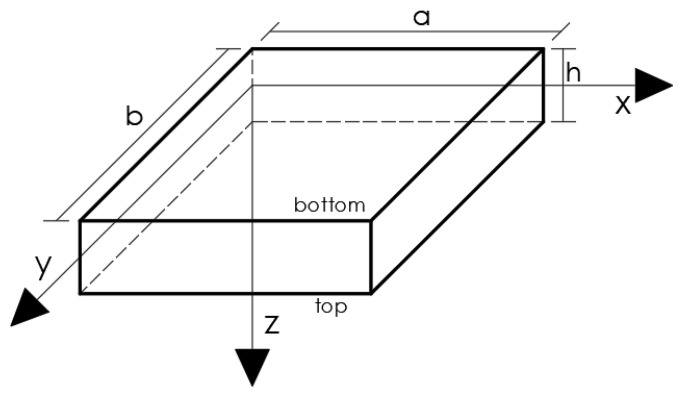
General laminate layout.

As show in Figure 1, consider a METE thin nanoplate with length *a*, width *b* and thickness *h*, in a Cartesian reference system (x,y,z).

The METE nanoplate is in a hygro-thermal environment and is subjected to an electric potential $V_{0}$ and to a magnetic potential $\Omega_{0}$ between the upper and lower surfaces. In this study, classic laminate plate theory is considered. We can define the displacement field of a generic point of the solid by means of the triad of displacement components *U*, *V*, *W*, which are functions of the coordinates (x,y,z) [10].

(1) $$\begin{array}{cc} \; & U\left(x,y,z,t\right)=u\left(x,y,t\right)-z\frac{\partial w}{\partial x}\\ & V\left(x,y,z,t\right)=v\left(x,y,t\right)-z\frac{\partial w}{\partial y}\\ & W(x,y,z,t)=w(x,y,t) \end{array}$$

where *u*, *v* and *w* are the displacements along the *x*, *y* and *z* axis of the point on the middle surface and ∂w/∂x and ∂w/∂y are the corresponding rotation. The constitutive equations for a METE material are:

(2) $$\begin{array}{cc} \sigma & =C\varepsilon -eE-qH-C\alpha \Delta T-C\beta \Delta H\\ D_{E} & =e^{⊤}\varepsilon +\xi E+\zeta H-p\Delta T-h\Delta H\\ B_{M} & =q^{⊤}\varepsilon +\zeta E+\chi H-\lambda \Delta T-\eta \Delta H \end{array}$$

in which σ is the classical stress vector, $D_{E}={\left[D_{x},\;D_{y},\;D_{z}\right]}^{⊤}$ and $B_{M}={\left[B_{x},\;B_{y},\;B_{z}\right]}^{⊤}$ are respectively the vector of stresses, electrical displacement and magnetic flux. ε, E and H are the vector of strain, electric field and magnetic field. C, ξ and χ represent the rigidity matrix, the electrical permittivity matrix and the magnetic permittivity matrix. Finally, e, q, ζ, p, λ, h and η are respectively the piezo-electric, piezo-magnetic, magneto-electro-elastic (MEE), pyro-electric, pyro-magnetic, hygro-electric and hygro-magnetic coefficients. For the stress plane state ($\sigma_{3}=0$) the matrices can be reduced by carrying out

(3) $$\varepsilon_{3}=-\frac{C_{13}}{C_{33}}\varepsilon_{1}-\frac{C_{23}}{C_{33}}\varepsilon_{2}+\frac{e_{33}}{C_{33}}E_{3}+\frac{q_{33}}{C_{33}}H_{3}+\frac{C_{13}}{C_{33}}\alpha_{1}\Delta T+\frac{C_{23}}{C_{33}}\alpha_{2}\Delta T+\frac{C_{13}}{C_{33}}\beta_{1}\Delta H+\frac{C_{23}}{C_{33}}\beta_{2}\Delta H$$

Therefore the constitutive equations can be rewritten as follows

(4) $$\begin{array}{cc} \sigma_{1} & =(C_{11}-\frac{C_{13}^{2}}{C_{33}})\varepsilon_{1}+(C_{12}-C_{13}\frac{C_{23}}{C_{33}})\varepsilon_{2}-(e_{31}-C_{13}\frac{e_{33}}{C_{33}})E_{3}-(q_{31}-C_{13}\frac{q_{33}}{C_{33}})H_{3}+\\ \; & -(C_{11}-\frac{C_{13}^{2}}{C_{33}})\alpha_{1}\Delta T-(C_{12}-C_{13}\frac{C_{23}}{C_{33}})\alpha_{2}\Delta T-(C_{11}-\frac{C_{13}^{2}}{C_{33}})\beta_{1}\Delta H-(C_{12}-C_{13}\frac{C_{23}}{C_{33}})\beta_{2}\Delta H\\ & =Q_{11}\varepsilon_{1}+Q_{12}\varepsilon_{2}-{\tilde{e}}_{31}E_{3}-{\tilde{q}}_{31}H_{3}-Q_{11}\alpha_{1}\Delta T-Q_{12}\alpha_{2}\Delta T-Q_{11}\beta_{1}\Delta H-Q_{12}\beta_{2}\Delta H \end{array}$$

similarly for $\sigma_{2}$ it will be

(5) $$\sigma_{2}=Q_{12}\varepsilon_{1}+Q_{22}\varepsilon_{2}-{\tilde{e}}_{32}E_{3}-{\tilde{q}}_{32}H_{3}-Q_{12}\alpha_{1}\Delta T-Q_{22}\alpha_{2}\Delta T-Q_{12}\beta_{1}\Delta H-Q_{22}\beta_{2}\Delta H$$

finally, $D_{z}$ can written

(6) $$\begin{array}{cc} D_{z} & =(e_{31}-e_{33}\frac{C_{13}}{C_{33}})\varepsilon_{1}+(e_{32}-e_{33}\frac{C_{23}}{C_{33}})\varepsilon_{2}+(\xi_{33}+\frac{e_{33}^{2}}{C_{33}})E_{3}+(\zeta_{33}+e_{33}\frac{q_{33}}{C_{33}})H_{3}+\\ & -(p_{3}-\frac{C_{13}}{C_{33}}\alpha_{1}-\frac{C_{23}}{C_{33}}\alpha_{2})\Delta T-(h_{3}-\frac{C_{13}}{C_{33}}\beta_{1}-\frac{C_{23}}{C_{33}}\beta_{2})\Delta H\\ & ={\tilde{e}}_{31}\varepsilon_{1}+{\tilde{e}}_{32}\varepsilon_{2}+{\tilde{\xi }}_{33}E_{3}+{\tilde{\zeta }}_{33}H_{3}-{\tilde{p}}_{3}\Delta T-{\tilde{h}}_{3}\Delta H \end{array}$$

and similarly for $B_{M,3}$ it will be

(7) $$B_{z}={\tilde{q}}_{31}\varepsilon_{1}+{\tilde{q}}_{32}\varepsilon_{2}+{\tilde{\zeta }}_{33}E_{3}+{\tilde{\chi }}_{33}H_{3}-{\tilde{\lambda }}_{3}\Delta T-{\tilde{\eta }}_{3}\Delta H$$

So it is possible to write

(8) $$\begin{array}{cc} \; & \tilde{e}=[\begin{array}{ccc} 0 & 0 & {\tilde{e}}_{31}\\ 0 & 0 & {\tilde{e}}_{32}\\ 0 & 0 & 0 \end{array}],\;\tilde{q}=[\begin{array}{ccc} 0 & 0 & {\tilde{q}}_{31}\\ 0 & 0 & {\tilde{q}}_{32}\\ 0 & 0 & 0 \end{array}],\;\tilde{\xi }=[\begin{array}{ccc} \xi_{1} & 0 & 0\\ 0 & \xi_{2} & 0\\ 0 & 0 & {\tilde{\xi }}_{3} \end{array}],\;\tilde{\chi }=[\begin{array}{ccc} \chi_{1} & 0 & 0\\ 0 & \chi_{2} & 0\\ 0 & 0 & {\tilde{\chi }}_{3} \end{array}],\;\\ & \tilde{\zeta }=[\begin{array}{ccc} \zeta_{1} & 0 & 0\\ 0 & \zeta_{2} & 0\\ 0 & 0 & {\tilde{\zeta }}_{3} \end{array}],\;p=\{\begin{array}{c} p_{1}\\ p_{2}\\ {\tilde{p}}_{3} \end{array}\},\;\lambda =\{\begin{array}{c} \lambda_{1}\\ \lambda_{2}\\ {\tilde{\lambda }}_{3} \end{array}\},\;h=\{\begin{array}{c} h_{1}\\ h_{2}\\ {\tilde{h}}_{3} \end{array}\},\;\eta =\{\begin{array}{c} \eta_{1}\\ \eta_{2}\\ {\tilde{\eta }}_{3} \end{array}\} \end{array}$$

By introducing second order strain gradient theory in the constitutive equations and by considering the mechanical properties variable with respect to the thickness direction we have (the dependency on the time *t* is omitted for the sake of simplicity)

(9) $$\begin{array}{cc} \sigma (x,y,z) & =\left(1-\ell^{2}\nabla^{2}\right)\left[\bar{Q}\left(z\right)\varepsilon -\tilde{e}\left(z\right)E-\tilde{q}\left(z\right)H\right]-\bar{Q}\left(z\right)\alpha \left(z\right)\Delta T-\bar{Q}\left(z\right)\beta \left(z\right)\Delta H\\ D_{E}\left(x,y,z\right) & =\left(1-\ell^{2}\nabla^{2}\right)\left[{\tilde{e}}^{⊤}\left(z\right)\varepsilon +\tilde{\xi }\left(z\right)E+\tilde{\zeta }\left(z\right)H\right]-p\left(z\right)\Delta T-h\left(z\right)\Delta H\\ B_{M}\left(x,y,z\right) & =\left(1-\ell^{2}\nabla^{2}\right)\left[{\tilde{q}}^{⊤}\left(z\right)\varepsilon +\tilde{\zeta }\left(z\right)E+\tilde{\chi }\left(z\right)H\right]-\lambda \left(z\right)\Delta T-\eta \left(z\right)\Delta H \end{array}$$

where *ℓ* is the nonlocal parameter and the operator $\nabla^{2}=\partial^{2}/\partial x^{2}+\partial^{2}/\partial y^{2}$ is the second order gradient operator. For the hygro-thermal loads a linear variation is considered along the thickness as

(10) $$\begin{array}{c} \Delta T=T_{0}+zT_{1}/h,\;\Delta H=H_{0}+zH_{1}/h \end{array}$$

## 3. Electric and Magnetic Potentials

To satisfy Maxwell’s equations [73] the electrical and magnetic potential are approximated along the thickness with a linear and cosinusoidal combination. The first amends for the open-circuit condition and the latter for the closed-circuit one

(11) $$\begin{array}{cc} \; & \Phi \left(x,y,z,t\right)=-\cos\frac{\pi z}{h}\phi \left(x,y,t\right)+\frac{2z}{h}V_{0}\\ & Y\left(x,y,z,t\right)=-\cos\frac{\pi z}{h}\gamma \left(x,y,t\right)+\frac{2z}{h}\Omega_{0} \end{array}$$

in which $V_{0}$ represents the difference in electrical potential between the two faces of the plate and $\Omega_{0}$ represents the difference in magnetic potential. The relationships between electric field and electric potential can be written in accordance with the above

(12) $$\begin{array}{cc} E_{x} & =-\frac{\partial \Phi }{\partial x}=\cos\frac{\pi z}{h}\frac{\partial \phi }{\partial x}\\ E_{y} & =-\frac{\partial \Phi }{\partial y}=\cos\frac{\pi z}{h}\frac{\partial \phi }{\partial y}\\ E_{z} & =-\frac{\partial \Phi }{\partial z}=-\frac{\pi }{h}\sin\frac{\pi z}{h}\phi -\frac{2}{h}V_{0} \end{array}$$

that in matrix notation can be rewritten in this form

(13) $$E=f_{E}D_{E}\phi +E_{0}$$

with

(14) $$f_{E}=\left[\begin{array}{ccc} \cos\frac{\pi z}{h} & 0 & 0\\ 0 & \cos\frac{\pi z}{h} & 0\\ 0 & 0 & -\frac{\pi }{h}\sin\frac{\pi z}{h} \end{array}\right],\;D_{E}=\left\{\begin{array}{c} \frac{\partial }{\partial x}\\ \frac{\partial }{\partial y}\\ 1 \end{array}\right\},\;E_{0}=\left\{\begin{array}{c} 0\\ 0\\ -\frac{2}{h}V_{0} \end{array}\right\}$$

Similarly for the magnetic field we can write as

(15) $$\begin{array}{cc} H_{x} & =-\frac{\partial Y}{\partial x}=\cos\frac{\pi z}{h}\frac{\partial \gamma }{\partial x}\\ H_{y} & =-\frac{\partial Y}{\partial y}=\cos\frac{\pi z}{h}\frac{\partial \gamma }{\partial y}\\ H_{z} & =-\frac{\partial Y}{\partial z}=-\frac{\pi }{h}\sin\frac{\pi z}{h}\gamma -\frac{2}{h}\Omega_{0} \end{array}$$

which in matrix notation becomes

(16) $$H=f_{H}D_{H}\gamma +H_{0}$$

with

(17) $$f_{H}=\left[\begin{array}{ccc} \cos\frac{\pi z}{h} & 0 & 0\\ 0 & \cos\frac{\pi z}{h} & 0\\ 0 & 0 & -\frac{\pi }{h}\sin\frac{\pi z}{h} \end{array}\right],\;D_{H}=\left\{\begin{array}{c} \frac{\partial }{\partial x}\\ \frac{\partial }{\partial y}\\ 1 \end{array}\right\},\;H_{0}=\left\{\begin{array}{c} 0\\ 0\\ -\frac{2}{h}\Omega_{0} \end{array}\right\}$$

## 4. Equations of Motion

The equations of motion are derived through Hamilton’s principle

(18) $$\begin{array}{c} \int_{t_{1}}^{t_{2}}\left(\delta H_{ent}+\delta V-\delta K\right)dt=0 \end{array}$$

Writing the variation of enthalpy $\delta H_{ent}$

(19) $$\begin{array}{cc} \delta H_{ent} & =\int_{A}\int_{-\frac{h}{2}}^{\frac{h}{2}}\{\sigma_{xx}\delta \varepsilon_{xx}+\sigma_{yy}\delta \varepsilon_{yy}+\sigma_{xy}\delta \gamma_{xy}\\ \; & -D_{x}\delta E_{x}-D_{y}\delta E_{y}-D_{z}\delta E_{z}-B_{x}\delta H_{x}-B_{y}\delta H_{y}-B_{z}\delta H_{z}\}\;dzdA \end{array}$$

by introducing the classical stress resultants $N_{xx}$, $N_{yy}$, $N_{xy}$ and $M_{xx}$, $M_{yy}$, $M_{xy}$ and the piezo and magneto resultants as

(20) $$\begin{array}{c} \left\{\begin{array}{c} D_{x}\\ D_{y}\\ D_{z} \end{array}\right\}=\int_{-\frac{h}{2}}^{\frac{h}{2}}f_{E}D_{E}\;dz,\;\left\{\begin{array}{c} B_{x}\\ B_{y}\\ B_{z} \end{array}\right\}=\int_{-\frac{h}{2}}^{\frac{h}{2}}f_{B}B_{M}\;dz \end{array}$$

The definition of the integrated elastic properties are given in the Appendix A in Equations (A1)–(A3). Thus, it is obtained

(21) $$\begin{array}{cc} \delta H_{ent}= & \int_{A}\{N_{xx}\left(\frac{\partial \delta u}{\partial x}+\frac{\partial w}{\partial x}\frac{\partial \delta w}{\partial x}\right)+N_{yy}\left(\frac{\partial \delta v}{\partial y}+\frac{\partial w}{\partial y}\frac{\partial \delta w}{\partial y}\right)\\ & +N_{xy}\left(\frac{\partial \delta u}{\partial y}+\frac{\partial \delta v}{\partial x}+\frac{\partial \delta w}{\partial y}\frac{\partial w}{\partial x}+\frac{\partial \delta w}{\partial x}\frac{\partial w}{\partial y}\right)+\\ & +M_{xx}\left(-\frac{\partial^{2}\delta w}{\partial x^{2}}\right)+M_{yy}\left(-\frac{\partial^{2}\delta w}{\partial y^{2}}\right)+M_{xy}\left(-2\frac{\partial^{2}\delta w}{\partial x\partial y}\right)+\\ & -\left(D_{x}\frac{\partial \delta \phi }{\partial x}+D_{y}\frac{\partial \delta \phi }{\partial y}+D_{x}\delta \phi \;+B_{x}\frac{\partial \delta \gamma }{\partial x}+B_{y}\frac{\partial \delta \gamma }{\partial y}+B_{z}\delta \gamma \;\right)\}dA \end{array}$$

Integrating by parts Equation (21) is obtained

(22) $$\begin{array}{cc} \delta H_{ent}= & \int_{A}\{\left(\frac{\partial N_{xx}}{\partial x}+\frac{\partial N_{xy}}{\partial y}\right)\delta u+\left(\frac{\partial N_{xy}}{\partial x}+\frac{\partial N_{yy}}{\partial y}\right)\delta v+[\frac{\partial }{\partial x}\left(N_{xx}\frac{\partial w}{\partial x}+N_{xy}\frac{\partial w}{\partial y}\right)\\ + & \frac{\partial }{\partial y}\left(N_{xy}\frac{\partial w}{\partial x}+N_{yy}\frac{\partial w}{\partial y}\right)+\frac{\partial^{2}M_{xx}}{\partial x^{2}}+\frac{\partial^{2}M_{yy}}{\partial y^{2}}+2\frac{\partial^{2}M_{xy}}{\partial x\partial y}]\delta w\;\\ - & \left(\frac{\partial D_{x}}{\partial x}+\frac{\partial D_{y}}{\partial y}+D_{z}\right)\delta \phi -\left(\frac{\partial B_{x}}{\partial x}+\frac{\partial B_{y}}{\partial y}+B_{z}\right)\delta \gamma \}dA\;+\\ - & \int_{\Gamma }\{\left(N_{xx}\;n_{x}+N_{xy}\;n_{y}\right)\delta u+\left(N_{xy}\;n_{x}+N_{yy}\;n_{y}\right)\delta v+[\left(N_{xx}n_{x}+N_{xy}n_{y}\right)\frac{\partial w}{\partial x}\\ + & \left(N_{xy}n_{x}+N_{yy}n_{y}\right)\frac{\partial w}{\partial y}+\left(\frac{\partial M_{xx}}{\partial x}+\frac{\partial M_{xy}}{\partial y}\right)n_{x}+\left(\frac{\partial M_{yy}}{\partial y}+\frac{\partial M_{xy}}{\partial x}\right)n_{y}]\delta w\\ - & \left(M_{xx}n_{x}+M_{xy}n_{y}\right)\frac{\partial \delta w}{\partial x}-\left(M_{xy}n_{x}+M_{yy}n_{y}\right)\frac{\partial \delta w}{\partial y}\\ - & \left(D_{x}\;n_{x}+D_{y}\;n_{y}\right)\delta \phi -\left(B_{x}\;n_{x}+B_{y}\;n_{y}\right)\delta \gamma \;\}d\Gamma \; \end{array}$$

The external work due to the external boundary loads (where mechanical, electrical and magnetic loads are neglected) can be written as

(23) $$\begin{array}{cc} \delta V= & \int_{\Gamma }\{\left({\hat{N}}_{xx}n_{x}+{\hat{N}}_{xy}n_{y}\right)\delta u\;+\left({\hat{N}}_{xy}n_{x}+{\hat{N}}_{yy}n_{y}\right)\delta v+\\ & \;-\left({\hat{M}}_{xx}n_{x}+{\hat{M}}_{xy}n_{y}\right)\frac{\partial \delta w}{\partial x}-\left({\hat{M}}_{xy}n_{x}+{\hat{M}}_{yy}n_{y}\right)\frac{\partial \delta w}{\partial y}\;+\left({\hat{Q}}_{x}n_{x}+{\hat{Q}}_{y}n_{y}\right)\delta w\}d\Gamma \end{array}$$

Variation of the Kinetic energy can be written as

(24) $$\begin{array}{cc} \delta K & =\int_{A}\{\left(-I_{0}\ddot{u}+I_{1}\frac{\partial \ddot{w}}{\partial x}\right)\delta u+\left(-I_{0}\ddot{v}+I_{1}\frac{\partial \ddot{w}}{\partial y}\right)\delta v\\ & +\left(-I_{0}\ddot{w}-I_{1}\frac{\partial \ddot{u}}{\partial x}-I_{1}\frac{\partial \ddot{v}}{\partial y}+I_{2}\frac{\partial \ddot{w}}{\partial x}+I_{2}\frac{\partial \ddot{w}}{\partial y}\right)\delta w\}dA+\\ \; & +\int_{\Gamma }\left\{I_{1}\ddot{u}n_{x}+I_{1}\ddot{v}n_{y}-I_{2}\frac{\partial \ddot{w}}{\partial x}n_{x}-I_{2}\frac{\partial \ddot{w}}{\partial y}\;n_{y}\right\}\delta w\;d\Gamma \end{array}$$

Introducing N(w) and P(w) as defined below

(25) $$\begin{array}{cc} N(w) & =\frac{\partial }{\partial x}\left(N_{xx}\frac{\partial w}{\partial x}\right)\delta w+\frac{\partial }{\partial y}\left(N_{yy}\frac{\partial w}{\partial y}\right)\delta w+\frac{\partial }{\partial y}\left(N_{xy}\frac{\partial w}{\partial x}\right)\delta w+\frac{\partial }{\partial x}\left(N_{xy}\frac{\partial w}{\partial y}\right)\delta w\\ P(w) & =\left(N_{xx}\frac{\partial w}{\partial x}+N_{xy}\frac{\partial w}{\partial y}\right)n_{x}+\left(N_{xy}\frac{\partial w}{\partial x}+N_{yy}\frac{\partial w}{\partial y}\right)n_{y} \end{array}$$

the motion equations can be written as follow

(26) $$\begin{array}{cc} \frac{\partial N_{xx}}{\partial x}+\frac{\partial N_{xy}}{\partial y} & =I_{0}\ddot{u}-I_{1}\frac{\partial \ddot{w}}{\partial x}\\ \frac{\partial N_{yy}}{\partial y}+\frac{\partial N_{xy}}{\partial x} & =I_{0}\ddot{v}-I_{1}\frac{\partial \ddot{w}}{\partial y}\\ \frac{\partial^{2}M_{xx}}{\partial x^{2}}+2\frac{\partial^{2}M_{xy}}{\partial x\partial y}+\frac{\partial^{2}M_{yy}}{\partial y^{2}}+N\left(w\right) & =I_{0}\ddot{w}+I_{1}\left(\frac{\partial \ddot{u}}{\partial x}+\frac{\partial \ddot{v}}{\partial y}\right)-I_{2}\left(\frac{\partial^{2}\ddot{w}}{\partial x^{2}}+\frac{\partial^{2}\ddot{w}}{\partial y^{2}}\right)\\ \frac{\partial D_{x}}{\partial x}+\frac{\partial D_{y}}{\partial y} & +D_{z}=0\\ \frac{\partial B_{x}}{\partial x}+\frac{\partial B_{y}}{\partial y} & +B_{z}=0 \end{array}$$

and relative boundary conditions become

(27) $$\begin{array}{cc} \delta u=0\; & \;\mathrm{or}\;\left(N_{xx}-{\hat{N}}_{xx}\right)n_{x}+\left(N_{xy}-{\hat{N}}_{xy}\right)n_{y}=0\\ \delta v=0\; & \;\mathrm{or}\;\left(N_{yy}-{\hat{N}}_{yy}\right)n_{y}+\left(N_{xy}-{\hat{N}}_{xy}\right)n_{x}=0\\ \delta w=0\; & \;\mathrm{or}\;\left(\frac{\partial M_{xx}}{\partial x}+\frac{\partial M_{xy}}{\partial y}-I_{1}\ddot{u}+I_{2}\frac{\partial \ddot{w}}{\partial x}\right)n_{x}+\\ \; & \;+\left(\frac{\partial M_{yy}}{\partial y}+\frac{\partial M_{xy}}{\partial x}-I_{1}\ddot{v}+I_{2}\frac{\partial \ddot{w}}{\partial y}\right)n_{y}+P\left(w\right)-\left({\hat{Q}}_{x}+{\hat{Q}}_{y}\right)=0\\ \frac{\partial \delta w}{\partial x}=0\; & \;\mathrm{or}\;\left(M_{xx}-{\hat{M}}_{xx}\right)n_{x}+\left(M_{xy}-{\hat{M}}_{xy}\right)n_{y}=0\\ \frac{\partial \delta w}{\partial y}=0\; & \;\mathrm{or}\;\left(M_{yy}-{\hat{M}}_{yy}\right)n_{y}+\left(M_{xy}-{\hat{M}}_{xy}\right)n_{x}=0\\ \delta \phi =0\; & \;\mathrm{or}\;D_{x}\;n_{x}+D_{y}\;n_{y}=0\\ \delta \gamma =0\; & \;\mathrm{or}\;B_{x}\;n_{x}+B_{y}\;n_{y}=0 \end{array}$$

## 5. Navier Solution

Analytical solution is obtained using Navier’s expansion. This type of solution allow to solve simply supported plate case. Navier expansion for the displacements take the form

(28) $$\left\{\begin{array}{c} u\\ v\\ w \end{array}\right\}=\sum_{m=1}^{M}\sum_{n=1}^{N}\left[\begin{array}{ccc} \cos\alpha x\sin\beta y & 0 & 0\\ 0 & \sin\alpha x\cos\beta y & 0\\ 0 & 0 & \sin\alpha x\sin\beta y \end{array}\right]\left\{\begin{array}{c} U_{mn}\\ V_{mn}\\ W_{mn} \end{array}\right\}$$

whereas, the electric and magnetic potentials are both approximated with a double sinusoidal trigonometric expansion.

(29) $$\phi =\sum_{m=1}^{M}\sum_{n=1}^{N}\sin\alpha x\sin\beta y\;\Phi_{mn},\;\gamma =\sum_{m=1}^{M}\sum_{n=1}^{N}\sin\alpha x\sin\beta y\;\Gamma_{mn}$$

### 5.1. Buckling Analysis

Replacing the displacement field in the motion equations and performing the derivates the algebraic system is obtained

(30) $$\left[\begin{array}{cccccc} {\hat{c}}_{11} & {\hat{c}}_{12} & {\hat{c}}_{14} & {\hat{c}}_{15} & {\hat{c}}_{13} & \\ {\hat{c}}_{12} & {\hat{c}}_{22} & {\hat{c}}_{24} & {\hat{c}}_{25} & {\hat{c}}_{23} & \\ {\hat{c}}_{14} & {\hat{c}}_{24} & {\hat{c}}_{44} & {\hat{c}}_{45} & {\hat{c}}_{34} & \\ {\hat{c}}_{15} & {\hat{c}}_{25} & {\hat{c}}_{45} & {\hat{c}}_{55} & {\hat{c}}_{35} & \\ {\hat{c}}_{13} & {\hat{c}}_{23} & {\hat{c}}_{34} & {\hat{c}}_{35} & {\hat{c}}_{33}+{\tilde{s}}_{33} & \end{array}\right]\left\{\begin{array}{c} U_{mn}\\ V_{mn}\\ \Phi_{mn}\\ \Gamma_{mn}\\ W_{mn} \end{array}\right\}=\left\{\begin{array}{c} 0\\ 0\\ 0\\ 0\\ 0 \end{array}\right\}$$

The coefficients ${\hat{c}}_{ij}$ and ${\tilde{s}}_{33}$ are defined in the Appendix A at Equation (A4). By introducing the quantities $N_{0}=-{\hat{N}}_{xx}$, $\kappa ={\hat{N}}_{yy}/{\hat{N}}_{xx}$ and $a_{mn}$ as

(31) $$\begin{array}{cc} \; & a_{mn}={\hat{c}}_{33}+\alpha^{2}\left({\hat{N}}_{xx}^{T}+{\hat{N}}_{xx}^{E}+{\hat{N}}_{xx}^{H}\right)+\beta^{2}\left({\hat{N}}_{yy}^{T}+{\hat{N}}_{yy}^{E}+{\hat{N}}_{yy}^{H}\right)\\ & \;-\left\{\begin{array}{cccc} {\hat{c}}_{13} & {\hat{c}}_{23} & {\hat{c}}_{34} & {\hat{c}}_{35} \end{array}\right\}{\left[\begin{array}{cccc} {\hat{c}}_{11} & {\hat{c}}_{12} & {\hat{c}}_{14} & {\hat{c}}_{15}\\ {\hat{c}}_{12} & {\hat{c}}_{22} & {\hat{c}}_{24} & {\hat{c}}_{25}\\ {\hat{c}}_{14} & {\hat{c}}_{24} & {\hat{c}}_{44} & {\hat{c}}_{45}\\ {\hat{c}}_{15} & {\hat{c}}_{25} & {\hat{c}}_{45} & {\hat{c}}_{55} \end{array}\right]}^{-1}\left\{\begin{array}{c} {\hat{c}}_{13}\\ {\hat{c}}_{23}\\ {\hat{c}}_{34}\\ {\hat{c}}_{35} \end{array}\right\} \end{array}$$

we can write the solution of the eigenvalue problem as

(32) $$N_{0}=\frac{a_{mn}}{\alpha^{2}+\kappa \beta^{2}}$$

The load that buckles the plate depends on *m* and *n* and in particular the critical load is the lowest of the buckling loads. The terms ${\hat{N}}_{xx}^{E}$, ${\hat{N}}_{yy}^{E}$, ${\hat{N}}_{xx}^{H}$, ${\hat{N}}_{yy}^{H}$ are defined below

(33) $${\hat{N}}_{xx}^{E}={\hat{N}}_{yy}^{E}=\int_{-h/2}^{h/2}{\bar{e}}_{31}\left(z\right)\frac{2V_{0}}{h}\;dz\;,\;{\hat{N}}_{xx}^{H}={\hat{N}}_{yy}^{H}=\int_{-h/2}^{h/2}{\bar{q}}_{31}\left(z\right)\frac{2\Omega_{0}}{h}\;dz$$

Note that the electric and magnetic in-plane loads have the same intensity since in the following applications the material is isotropic in-plane and anisotropic out-of-plane.

### 5.2. Thermal Free Vibration

In this paragraph it will be treated the problem of free vibrations of the FG plate simply supported. For this problem it is necessary to rewrite the solving system in a homogeneous form, and the rotational inertia $I_{1}$ are neglected. The system becomes then

(34) $$\begin{array}{c} \left(\left[\begin{array}{ccccc} {\hat{c}}_{11} & {\hat{c}}_{12} & {\hat{c}}_{13} & {\hat{c}}_{14} & {\hat{c}}_{15}\\ {\hat{c}}_{12} & {\hat{c}}_{22} & {\hat{c}}_{23} & {\hat{c}}_{24} & {\hat{c}}_{25}\\ {\hat{c}}_{13} & {\hat{c}}_{23} & {\hat{c}}_{33} & {\hat{c}}_{34} & {\hat{c}}_{35}\\ {\hat{c}}_{14} & {\hat{c}}_{24} & {\hat{c}}_{34} & {\hat{c}}_{44} & {\hat{c}}_{45}\\ {\hat{c}}_{15} & {\hat{c}}_{25} & {\hat{c}}_{35} & {\hat{c}}_{45} & {\hat{c}}_{55} \end{array}\right]-\omega^{2}\left[\begin{array}{ccccc} {\hat{m}}_{11} & 0 & 0 & 0 & 0\\ 0 & {\hat{m}}_{22} & 0 & 0 & 0\\ 0 & 0 & {\hat{m}}_{33} & 0 & 0\\ 0 & 0 & 0 & 0 & 0\\ 0 & 0 & 0 & 0 & 0 \end{array}\right]\right)\left\{\begin{array}{c} U_{mn}\\ V_{mn}\\ W_{mn}\\ \Phi_{mn}\\ \Gamma_{mn} \end{array}\right\}=\left\{\begin{array}{c} 0\\ 0\\ 0\\ 0\\ 0 \end{array}\right\} \end{array}$$

with ${\hat{m}}_{11}={\hat{m}}_{22}=I_{0}$ e ${\hat{m}}_{33}=I_{0}+I_{2}\left(\alpha^{2}+\beta^{2}\right)$. This system can then be rewritten in a more compact matrix form as follows

(35) $$\left(\left[\begin{array}{cc} K_{uu} & K_{u\phi }\\ K_{\phi u} & K_{\phi \phi } \end{array}\right]-\omega^{2}\left[\begin{array}{cc} M_{uu} & 0\\ 0 & 0 \end{array}\right]\right)\left\{\begin{array}{c} U\\ \phi \end{array}\right\}=\left\{\begin{array}{c} 0\\ 0 \end{array}\right\}$$

Rewriting the matrix system by applying static condensation we get

(36) $$(\bar{K}-\omega^{2}M_{uu})\;U=\;0$$

where $\bar{K}$ is

(37) $$\bar{K}=\left(K_{uu}-K_{u\phi }{\left(K_{\phi \phi }\right)}^{-1}K_{\phi u}\right)$$

and U represents the ways of vibrating and ω natural frequencies.

## 6. Numerical Results

In this paper for the numerical solution has been considered a FG nanoplate composed of CoFe${}_{2}$O${}_{4}$ and BaTiO${}_{3}$, the properties of the materials are shown in Table 1. Since it has not been possible to find in literature the hygrometric coefficients of the materials, the applications presented foresee only the case of thermal environment.

The variation of the material properties along the thickness is regulated by the following relationship

(38) $$P\left(z\right)=\left(P_{t}-P_{b}\right){\left(\frac{z}{h}+\frac{1}{2}\right)}^{p_{n}}+P_{b}$$

where $P_{t}$ and $P_{b}$ represent the properties of the material placed on the top and bottom of the plate, respectively.

Note that if $p_{n}=0$ the plate will be composed entirely of the material of the top side while if $p_{n}\to \infty$ the plate will be composed entirely of the material of the bottom side.

### 6.1. Buckling

In the following applications the values of the critical load will be presented in dimensionless form through the following relationship

(39) $${\bar{N}}_{cr}=\frac{N_{cr}a^{2}}{C_{11,m}h^{3}}$$

where $C_{11,m}$ is the average stiffness value of the two materials. As a first result the comparison with Li [74], Park and Han [75] is reported. The plate considered is isotropic with a/h=1000 and the properties of the material used are as reported in the cited works (which are obtained as average values of two isotropic constituents, not as a functionally graded composite).

**Figure 2:**
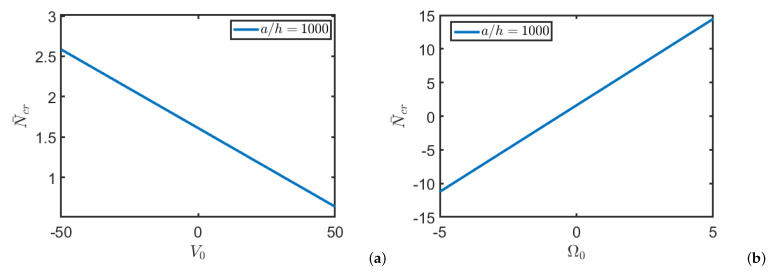
Critical load ${\bar{N}}_{cr}$ of a square Functionally Graded (FG) nanoplate for different values of electric potential $V_{0}$ (**a**) and magnetic potential $\Omega_{0}$ (**b**).

Figure 2 shows the critical buckling load by varying the electric and magnetic potentials. The present results agree well with the ones presented in the mentioned papers [74,75]. It is emphasized that the slight difference in the results is due to the fact that in the cited studies the potential is approximated using three contributions: one parabolic, one linear and one constant unlike the present study in which the potentials are approximated with a cosine function and a linear part. In addition, in the cited studies, the in-plane components $E_{x}$, $E_{y}$, $H_{x}$ and $H_{y}$ of electric and magnetic fields are null.

**Figure 3:**
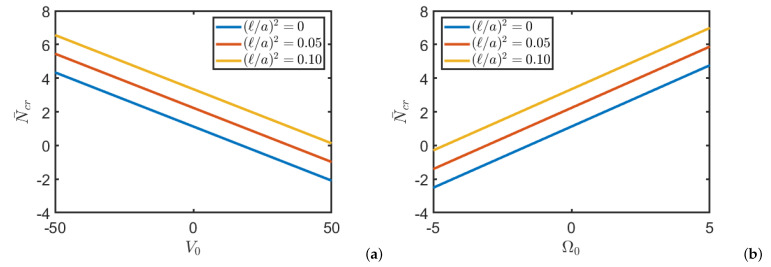
Critical load ${\bar{N}}_{cr}$ of a square FG nanoplate for different values of electric potential $V_{0}$ (**a**) and magnetic potential $\Omega_{0}$ (**b**), for different values of non local parameter ${\left(\ell /a\right)}^{2}$.

**Figure 4:**
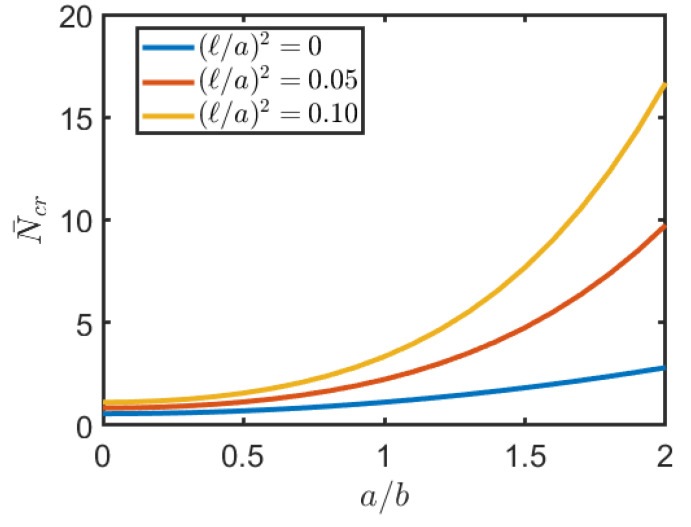
Critical load ${\bar{N}}_{cr}$ of square FG nanoplate for different values of ratio a/b and different values of non local parameter ${\left(\ell /a\right)}^{2}$.

In the applications below a/h=1000 and $n_{p}=1$ are always considered. Table 2 shows the dimensionless critical load of a square plate FG for different values of externally applied potentials and non-local parameter. It can be seen that as the non-local parameter increases, the value of the critical load increases. It can also be seen that by increasing the magnetic potential the critical load increases while the electric potential has the opposite effect. This last phenomenon is also clearly visible in Figure 3, where the value of the critical load is reported as the external potentials applied vary and for different values of the non-local parameter. Finally it is remarked that for same values of the electric and magnetic potentials the critical load takes a negative value, thus, buckling occurs for traction loads instead of compression. Figure 4 shows the dimensional critical load when the aspect ratio varies and for different values of the non-local parameter. As expected the critical load increases as the plane becomes of rectangular shape and as the nonlocal parameter increases.

### 6.2. Thermal Free Vibration

As first application for the free vibration of piezo-electro-magnetic-thermal plates a comparison with [76] is reported (Table 3). The plate is composed of BaTiO${}_{3}$/CoFe${}_{2}$O${}_{4}$ and is of rectangular shape with a=2 and b=1, ratio h/a=0.1 and properties vary linearly along the thickness ($n_{p}=1$). The results are written in dimensionless form through the following formula

(40) $$\bar{\omega }=\omega \left(\frac{a^{2}}{h}\right)\sqrt{\frac{\rho }{C_{11}}}$$

where ρ and $C_{11}$ respectively represent the density and the (1,1) position element of the material stiffness matrix on the underside of the plate. It should be noted that in the study just mentioned the Mindlin’s moderately thick plates theory (FSDT) is used, and so the results deviate slightly and this difference increases as the vibration mode increases as the effects of shear become more relevant.

Table 4 shows a comparison with article [57] for a thick magneto-electro-elastic square plate. The thickness of the plate is constant and the laminae all have the same thickness. The properties of the material are those reported in Table 1, except the density which is assumed constant for the two materials and equal to 1600 kg/m${}^{3}$. The values calculated in this study differ slightly from those in the literature because in [57] a third order plate theory is used while in this study it is used thin plate theory.

Table 5 analyzes the influence of the non-local parameter on the natural frequencies of a square plate with a=b=1 m and ratio a/h=100 and $n_{p}=1$. The results show an increase in natural frequencies, due to the stiffening of the plate, as the non-local parameter increases.

**Figure 5:**
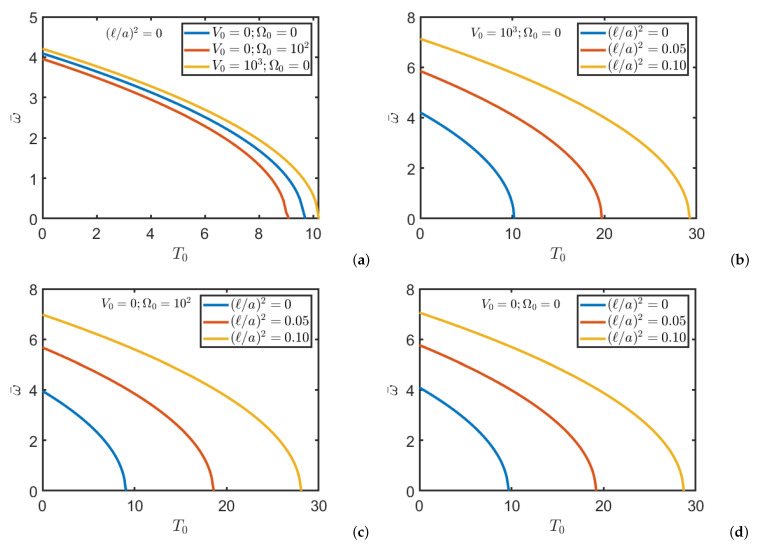
Natural frequencies $\bar{\omega }$ of a simply supported square FG nanoplate composed by BaTiO${}_{3}$/CoFe${}_{2}$O${}_{4}$ to vary of temperature $T_{0}$ and for different values of magnetic and electric potentials and non-local parameter: (**a**) local configuration (ℓ=0); (**b**) nonlocal configuration with $V_{0}\neq 0$; (**c**) nonlocal configuration with $\Omega_{0}\neq 0$; (**d**) nonlocal configuration with $V_{0}=\Omega_{0}=0$.

Figure 5 shows the influence of temperature and externally applied potentials on the natural frequency of a nanoplate composed by BaTiO${}_{3}$/CoFe${}_{2}$O${}_{4}$. The plate considered is a simply supported square plate and the thickness ratio is a/h=100. In particular, the critical temperature of each structure can be identified when the frequency becomes zero.

## 7. Conclusions

In this paper the dynamic and buckling problems of METE nanoplates have been analyzed. In particular, the interest focused on the coupling of magnet-electro-thermo-elastic effects and the influence that the external potentials applied to the plate have on the critical load and natural vibration frequencies. Through Hamilton’s principle motion equations for FG METE thin plates are derived and analytical solution using Navier method is obtained. The materials that have been used in the simulations are BaTiO${}_{3}$ and CoFe${}_{2}$O${}_{4}$ and the properties of the materials used are reported in the article. The results show that increasing the non-local parameter increases the critical load and natural vibration frequencies. For external potentials instead it was seen that the critical load increases with the increase of the negative electrical potential and the positive magnetic potential. Finally, from the graphs of the natural frequency of vibration it can be seen that the frequencies tend to increase by subjecting the plate to a positive magnetic potential and to decrease by subjecting it to a positive electrical potential. For what concerns the temperature instead we see how an increase of the latter leads to a reduction of the natural frequencies.

## Figures and Tables

**Table 1:** Piezo-electro-magnetic-thermal properties of BaTiO${}_{3}$ and CoFe${}_{2}$O${}_{4}$.

		BaTiO${}_{3}$	CoFe${}_{2}$O${}_{4}$
$C_{11}$	[GPa]	166	286
$C_{22}$		166	286
$C_{33}$		162	269.5
$C_{13}$		78	170.5
$C_{23}$		78	170.5
$C_{12}$		77	173
$C_{44}$		43	45.3
$C_{55}$		43	45.3
$C_{66}$		44.5	56.5
$e_{31}$	[C/m${}^{2}$]	−4.4	0
$e_{32}$		−4.4	0
$e_{33}$		18.6	0
$q_{31}$	[N/A·m]	0	580.3
$q_{32}$		0	580.3
$q_{33}$		0	699.7
$\xi_{11}$	[$10^{-9}$C${}^{2}$/N·m${}^{2}$]	11.2	0.08
$\xi_{22}$		11.2	0.08
$\xi_{33}$		12.6	0.093
$\zeta_{11}=\zeta_{22}=\zeta_{33}$	[s/m]	0	0
$\chi_{11}$	[$10^{-6}$N·s${}^{2}$/C]	5	−590
$\chi_{22}$		5	−590
$\chi_{33}$		10	157
$p_{11}=p_{22}$	[$10^{-7}$ C/m${}^{2}$K]	0	0
$p_{33}$		−11.4	0
$\lambda_{11}=\lambda_{22}$	[$10^{-5}$ Wb/m${}^{2}$K]	0	0
$\lambda_{33}$		0	−36.2
$\alpha_{1}=\alpha_{2}$	[$10^{-6}$K${}^{-1}$]	15.8	10
ρ	[kg/m${}^{3}$]	5300	5800

**Table 2:** Dimensionless critical load ${\bar{N}}_{cr}$ of a square FG plate composed of BaTiO${}_{3}$/CoFe${}_{2}$O${}_{4}$ for different electric and magnetic potentials and nonlocal parameter ${\left(\ell /a\right)}^{2}$. (κ=1,(m,n)=(1,1)).

		$V_{0}$ [V]				
${\left(\ell /a\right)}^{2}$	$\Omega_{0}$ [A]	−5	−2.5	0	2.5	5
0.00	1	2.1733	2.0124	1.8516	1.6907	1.5299
	0	1.4456	1.2848	1.1239	0.9631	0.8022
	−1	0.7180	0.5572	0.3963	0.2355	0.0746
0.05	1	3.2825	3.1217	2.9608	2.8000	2.6391
	0	2.5549	2.3940	2.2332	2.0723	1.9115
	−1	1.8273	1.6664	1.5056	1.3447	1.1838
0.10	1	4.3918	4.2309	4.0701	3.9092	3.7484
	0	3.6642	3.5033	3.3424	3.1816	3.0207
	−1	2.9365	2.7757	2.6148	2.4540	2.2931

**Table 3:** Dimensionless natural frequencies $\bar{\omega }$ of a simply supported rectangular FG plate composed of BaTiO${}_{3}$/CoFe${}_{2}$O${}_{4}$.

	Ref. [76]	Present
1	9.525	10.0244
2	28.762	32.5716
3	50.966	66.2842
4	131.186	104.0065
5	139.106	129.6477

**Table 4:** Natural frequencies (rad/s) of a simply supported square plate (a=1 m; h=0.3 m). B = BaTiO${}_{3}$; F = CoFe${}_{2}$O${}_{4}$.

Mode	Ref. [57] ${\left(\ell /a\right)}^{2}=0$			
	B	F	B/F/B	F/B/F
1	12,863.98	15,185.24	13,024.78	15,043.32
2	25,106.78	28,177.03	25,401.26	27,880.80
	Present ${\left(\ell /a\right)}^{2}=0$			
1	15,044.28	17,253.16	15,281.37	17,159.99
2	34,945.88	39,415.58	28,264.36	39,871.43
	Present ${\left(\ell /a\right)}^{2}=0.05$			
1	21,065.74	24,351.13	20,932.93	24,069.44
2	63,337.77	73,727.92	62,619.25	72,511.91
	Present ${\left(\ell /a\right)}^{2}=0.10$			
1	25,723.93	29,805.02	25,499.40	29,410.18
2	82,655.46	96,547.86	81,654.82	94,727.82

**Table 5:** Natural frequencies $\bar{\omega }$ of a simply supported square FG nanoplate composed by BaTiO${}_{3}$/CoFe${}_{2}$O${}_{4}$.

	${\left(\ell /a\right)}^{2}$		
	**0**	**0.05**	**0.10**
1	4.0913	5.7671	7.0554
2	10.2270	19.0434	24.9141
3	20.4499	49.8179	67.4200
4	34.7550	106.4926	146.5385
5	53.1353	197.6005	274.3517

## Data Availability

Data sharing not applicable.

## Appendix A

Integrating last two integrals of Equation (22) along the thickness the following quantities can be defined

(A1) $$\begin{array}{c} A_{E}^{f}=\int_{-\frac{h}{2}}^{\frac{h}{2}}\tilde{e}f_{E}\;dz,\;A_{E}=\int_{-\frac{h}{2}}^{\frac{h}{2}}\tilde{e}\;dz,\;B_{E}^{f}=\int_{-\frac{h}{2}}^{\frac{h}{2}}\tilde{e}f_{E}\;z\;dz\;,\;B_{E}=\int_{-\frac{h}{2}}^{\frac{h}{2}}\tilde{e}\;z\;dz\\ A_{H}^{f}=\int_{-\frac{h}{2}}^{\frac{h}{2}}\tilde{q}f_{H}\;dz,\;A_{H}=\int_{-\frac{h}{2}}^{\frac{h}{2}}\tilde{q}\;dz,\;B_{H}^{f}=\int_{-\frac{h}{2}}^{\frac{h}{2}}\tilde{q}f_{H}\;z\;dz,\;B_{H}=\int_{-\frac{h}{2}}^{\frac{h}{2}}\tilde{q}\;z\;dz \end{array}$$

(A2) $$\begin{array}{c} A_{\xi }^{f}=\int_{-\frac{h}{2}}^{\frac{h}{2}}f_{E}^{⊤}\tilde{\xi }dz,\;B_{\xi }^{f}=\int_{-\frac{h}{2}}^{\frac{h}{2}}f_{E}^{⊤}\tilde{\xi }f_{E}dz,\;A_{\zeta }^{f}=\int_{-\frac{h}{2}}^{\frac{h}{2}}f_{E}^{⊤}\tilde{\zeta }dz,\;B_{\zeta }^{f}=\int_{-\frac{h}{2}}^{\frac{h}{2}}f_{E}^{⊤}\tilde{\zeta }f_{H}dz\\ A_{p}=\int_{-\frac{h}{2}}^{\frac{h}{2}}f_{E}^{⊤}pdz,\;B_{p}=\int_{-\frac{h}{2}}^{\frac{h}{2}}f_{E}^{⊤}pz\;dz,\;A_{h}=\int_{-\frac{h}{2}}^{\frac{h}{2}}f_{E}^{⊤}hdz,\;B_{h}=\int_{-\frac{h}{2}}^{\frac{h}{2}}f_{E}^{⊤}hz\;dz \end{array}$$

(A3) $$\begin{array}{c} A_{\zeta }^{f}=\int_{-\frac{h}{2}}^{\frac{h}{2}}f_{H}^{⊤}\tilde{\zeta }dz,\;B_{\zeta }^{f}=\int_{-\frac{h}{2}}^{\frac{h}{2}}f_{H}^{⊤}\tilde{\zeta }f_{E}dz,\;A_{\chi }^{f}=\int_{-\frac{h}{2}}^{\frac{h}{2}}f_{H}^{⊤}\tilde{\chi }dz,\;B_{\chi }^{f}=\int_{-\frac{h}{2}}^{\frac{h}{2}}f_{H}^{⊤}\tilde{\chi }f_{H}dz\\ A_{\lambda }=\int_{-\frac{h}{2}}^{\frac{h}{2}}f_{H}^{⊤}\lambda dz,\;B_{\lambda }=\int_{-\frac{h}{2}}^{\frac{h}{2}}f_{H}^{⊤}\lambda z\;dz,\;A_{\eta }=\int_{-\frac{h}{2}}^{\frac{h}{2}}f_{H}^{⊤}\eta dz,\;B_{\eta }=\int_{-\frac{h}{2}}^{\frac{h}{2}}f_{H}^{⊤}\eta z\;dz \end{array}$$

Considering what reported in Equations (A1)–(A3) it can be write ${\hat{c}}_{ij}$ coefficients as follows

(A4) $$\begin{array}{cc} {\hat{c}}_{11} & =\alpha^{2}A_{11}+\beta^{2}A_{66}+\ell^{2}\left[\alpha^{4}A_{11}+\alpha^{2}\beta^{2}\left(A_{11}+A_{66}\right)+\beta^{4}A_{66}\right]\\ {\hat{c}}_{12} & =\alpha \beta \left(A_{12}+A_{66}\right)+\ell^{2}\left[\alpha^{3}\beta \left(A_{12}+A_{66}\right)+\alpha \beta^{3}\left(A_{12}+A_{66}\right)\right]\\ {\hat{c}}_{13} & =-\alpha^{3}B_{11}-\alpha \beta^{2}\left(B_{12}+2B_{66}\right)\\ & \;-\ell^{2}\left[\alpha^{5}B_{11}+\alpha^{3}\beta^{2}\left(B_{12}+B_{11}+2B_{66}\right)+\alpha \beta^{4}\left(B_{12}+2B_{66}\right)\right]\\ {\hat{c}}_{22} & =\beta^{2}A_{22}+\alpha^{2}A_{66}+\ell^{2}\left[\alpha^{2}\beta^{2}\left(A_{22}+A_{66}\right)+\alpha^{4}A_{66}+\beta^{4}A_{22}\right]\\ {\hat{c}}_{23} & =-\alpha^{2}\beta \left(B_{12}+2B_{66}\right)-\beta^{3}B_{22}\\ & \;-\ell^{2}\left[\alpha^{4}\beta \left(B_{12}+2B_{66}\right)+\alpha^{2}\beta^{3}\left(B_{22}+B_{12}+2B_{66}\right)+\beta^{5}B_{22}\right]\\ {\hat{c}}_{33} & =\alpha^{4}D_{11}+2\alpha^{2}\beta^{2}\left(D_{12}+2D_{66}\right)+\beta^{4}D_{22}+\ell^{2}[\alpha^{6}D_{11}+\alpha^{4}\beta^{2}\left(D_{11}+2D_{12}+4D_{66}\right)\\ & \;\;+\alpha^{2}\beta^{4}\left(D_{22}+2D_{12}+4D_{66}\right)+\beta^{6}D_{22}]\\ {\hat{c}}_{14} & =-\alpha A_{E,13}^{f}+\ell^{2}\left[-\alpha^{3}A_{E,13}^{f}-\alpha \beta^{2}A_{E,13}^{f}\right]\\ {\hat{c}}_{15} & =-\alpha A_{H,13}^{f}+\ell^{2}\left[-\alpha^{3}A_{H,13}^{f}-\alpha \beta^{2}A_{H,13}^{f}\right]\\ {\hat{c}}_{24} & =-\beta A_{E,23}^{f}+\ell^{2}\left[-\beta^{3}A_{E,23}^{f}-\alpha^{2}\beta A_{E,23}^{f}\right]\\ {\hat{c}}_{25} & =-\beta A_{H,23}^{f}+\ell^{2}\left[-\beta^{3}A_{H,23}^{f}-\alpha^{2}\beta A_{H,23}^{f}\right]\\ {\hat{c}}_{34} & =\alpha^{2}B_{E,13}^{f}+\beta^{2}B_{E,23}^{f}+\ell^{2}\left[\alpha^{4}B_{E,13}^{f}+\alpha^{2}\beta^{2}\left(B_{E,13}^{f}+B_{E,23}^{f}\right)+\beta^{4}B_{E,23}^{f}\right]\\ {\hat{c}}_{35} & =\alpha^{2}B_{H,13}^{f}+\beta^{2}B_{H,23}^{f}+\ell^{2}\left[\alpha^{4}B_{H,13}^{f}+\alpha^{2}\beta^{2}\left(B_{H,13}^{f}+B_{H,23}^{f}\right)+\beta^{4}B_{H,23}^{f}\right]\\ {\hat{c}}_{44} & =-B_{\xi ,33}^{f}-\alpha^{2}B_{\xi ,11}^{f}-\beta^{2}B_{\xi ,22}^{f}\\ & \;-\ell^{2}\left[\alpha^{4}B_{\xi ,11}^{f}+\alpha^{2}\beta^{2}\left(B_{\xi ,11}^{f}+B_{\xi ,22}^{f}\right)+\beta^{4}B_{\xi ,22}^{f}+\alpha^{2}B_{\xi ,33}^{f}+\beta^{2}B_{\xi ,33}^{f}\right]\\ {\hat{c}}_{45} & =-B_{\zeta ,33}^{fE}-\alpha^{2}B_{\zeta ,11}^{fE}-\beta^{2}B_{\zeta ,22}^{fE}\\ & \;-\ell^{2}\left[\alpha^{4}B_{\zeta ,11}^{fE}+\alpha^{2}\beta^{2}\left(B_{\zeta ,11}^{fE}+B_{\zeta 22}^{fE}\right)+\beta^{4}B_{\zeta ,22}^{fE}+\alpha^{2}B_{\zeta ,33}^{fE}+\beta^{2}B_{\zeta ,33}^{fE}\right]\\ {\hat{c}}_{54} & =-B_{\zeta ,33}^{fH}-\alpha^{2}B_{\zeta ,11}^{fH}-\beta^{2}B_{\zeta ,22}^{fH}\\ & \;-\ell^{2}\left[\alpha^{4}B_{\zeta ,11}^{fH}+\alpha^{2}\beta^{2}\left(B_{\zeta ,11}^{fH}+B_{\zeta ,22}^{fH}\right)+\beta^{4}B_{\zeta ,22}^{fH}+\alpha^{2}B_{\zeta ,33}^{fH}+\beta^{2}B_{\zeta ,33}^{fH}\right]\\ {\hat{c}}_{55} & =-B_{\chi ,33}^{f}-\alpha^{2}B_{\chi ,11}^{f}-\beta^{2}B_{\chi 22}^{f}\\ & \;-\ell^{2}\left[\alpha^{4}B_{\chi ,11}^{f}+\alpha^{2}\beta^{2}\left(B_{\chi ,11}^{f}+B_{\chi ,22}^{f}\right)+\beta^{4}B_{\chi ,22}^{f}+\alpha^{2}B_{\chi ,33}^{f}+\beta^{2}B_{\chi ,33}^{f}\right]\\ {\tilde{s}}_{33} & =\alpha \left({\hat{N}}_{xx}+{\hat{N}}_{xx}^{T}+{\hat{N}}_{xx}^{E}+{\hat{N}}_{xx}^{H}\right)+\beta \left({\hat{N}}_{yy}+{\hat{N}}_{yy}^{T}+{\hat{N}}_{yy}^{E}+{\hat{N}}_{yy}^{H}\right) \end{array}$$

In case the electrical and magnetic potentials have the same through-the-thickness expansion, as in the present study, $B_{\zeta }^{fE}=B_{\zeta }^{fH}=B_{\zeta }^{f}$, hence, ${\hat{c}}_{45}={\hat{c}}_{54}$.

## References

[1] Saji V.S., Choe H.C., Yeung K.W. (2010). Nanotechnology in biomedical applications: A review. Int. J. Nano-Biomater..

[2] Berman D., Krim J. (2013). Surface science, MEMS and NEMS: Progress and opportunities for surface science research performed on, or by, microdevices. Prog. Surf. Sci..

[3] Bhushan B. (2007). Nanotribology and nanomechanics of MEMS/NEMS and BioMEMS/BioNEMS materials and devices. Microelectron. Eng..

[4] Ekinci K.L., Roukes M.L. (2005). Nanoelectromechanical systems. Rev. Sci. Instruments.

[5] Barretta R., Fabbrocino F., Luciano R., de Sciarra F.M., Ruta G. (2020). Buckling loads of nano-beams in stress-driven nonlocal elasticity. Mech. Adv. Mater. Struct..

[6] Bonanni A., del Valle M. (2010). Use of nanomaterials for impedimetric DNA sensors: A review. Anal. Chim. Acta.

[7] Wu W. (2017). Inorganic nanomaterials for printed electronics: A review. Nanoscale.

[8] Gohardani O., Elola M.C., Elizetxea C. (2014). Potential and prospective implementation of carbon nanotubes on next generation aircraft and space vehicles: A review of current and expected applications in aerospace sciences. Prog. Aerosp. Sci..

[9] Singh T. (2014). A review of nanomaterials in civil engineering works. Inter. J. Struct. Civ. Eng. Res..

[10] Amabili M. (2018). Nonlinear Mechanics of Shells and Plates in Composite, Soft and Biological Materials.

[11] Lakes R. (1986). Experimental microelasticity of two porous solids. Int. J. Solids Struct..

[12] Stölken J., Evans A. (1998). A microbend test method for measuring the plasticity length scale. Acta Mater..

[13] Mancusi G., Fabbrocino F., Feo L., Fraternali F. (2017). Size effect and dynamic properties of 2D lattice materials. Compos. Part B Eng..

[14] Fabbrocino F., Carpentieri G. (2017). Three-dimensional modeling of the wave dynamics of tensegrity lattices. Compos. Struct..

[15] Eringen A., Edelen D. (1972). On nonlocal elasticity. Int. J. Eng. Sci..

[16] Trovalusci P., Sadowski T., Trovalusci P. (2014). Molecular Approaches for Multifield Continua: Origins and Current Developments. Multiscale Modeling of Complex Materials.

[17] Eringen A.C. (1983). On differential equations of nonlocal elasticity and solutions of screw dislocation and surface waves. J. Appl. Phys..

[18] Aifantis E. (2003). Update on a class of gradient theories. Mech. Mater..

[19] Meenen J., Altenbach H., Eremeyev V., Naumenko K. (2011). A Variationally Consistent Derivation of Microcontinuum Theories. Adv. Struct. Mater..

[20] Mindlin R., Eshel N. (1968). On first strain-gradient theories in linear elasticity. Int. J. Solids Struct..

[21] Karami B., Janghorban M., Rabczuk T. (2019). Static analysis of functionally graded anisotropic nanoplates using nonlocal strain gradient theory. Compos. Struct..

[22] Eremeyev V., Altenbach H., Altenbach H., Mikhasev G.I. (2015). On the Direct Approach in the Theory of Second Gradient Plates. Shell and Membrane Theories in Mechanics and Biology.

[23] Bacciocchi M., Fantuzzi N., Ferreira A. (2020). Conforming and nonconforming laminated finite element Kirchhoff nanoplates in bending using strain gradient theory. Comput. Struct..

[24] Barretta R., Feo L., Luciano R., Marotti de Sciarra F., Penna R. (2016). Functionally graded Timoshenko nanobeams: A novel nonlocal gradient formulation. Compos. Part B Eng..

[25] Sahmani S., Aghdam M.M., Rabczuk T. (2018). Nonlinear bending of functionally graded porous micro/nano-beams reinforced with graphene platelets based upon nonlocal strain gradient theory. Compos. Struct..

[26] Jamalpoor A., Hosseini M. (2015). Biaxial buckling analysis of double-orthotropic microplate-systems including in-plane magnetic field based on strain gradient theory. Compos. Part B Eng..

[27] Apuzzo A., Barretta R., Faghidian S., Luciano R., Marotti de Sciarra F. (2018). Free vibrations of elastic beams by modified nonlocal strain gradient theory. Int. J. Eng. Sci..

[28] Yang F., Chong A., Lam D., Tong P. (2002). Couple stress based strain gradient theory for elasticity. Int. J. Solids Struct..

[29] Mühlhaus H., Oka F. (1996). Dispersion and wave propagation in discrete and continuous models for granular materials. Int. J. Solids Struct..

[30] Leonetti L., Greco F., Trovalusci P., Luciano R., Masiani R. (2018). A multiscale damage analysis of periodic composites using a couple-stress/Cauchy multidomain model: Application to masonry structures. Compos. Part B Eng..

[31] Farajpour A., Howard C.Q., Robertson W.S. (2020). On size-dependent mechanics of nanoplates. Int. J. Eng. Sci..

[32] Barretta R., Faghidian S.A., Marotti de Sciarra F. (2019). Stress-driven nonlocal integral elasticity for axisymmetric nano-plates. Int. J. Eng. Sci..

[33] Trovalusci P., Bellis M.D., Ostoja-Starzewski M. (2016). A Statistically-Based Homogenization Approach for Particle Random Composites as Micropolar Continua. Adv. Struct. Mater..

[34] Reccia E., De Bellis M.L., Trovalusci P., Masiani R. (2018). Sensitivity to material contrast in homogenization of random particle composites as micropolar continua. Compos. Part B Eng..

[35] Fantuzzi N., Leonetti L., Trovalusci P., Tornabene F. (2018). Some Novel Numerical Applications of Cosserat Continua. Int. J. Comput. Methods.

[36] Rega G., Saetta E., Settimi V. (2020). Modeling and nonlinear dynamics of thermomechanically coupled composite plates. Int. J. Mech. Sci..

[37] Kim J.Y. (2011). Micromechanical analysis of effective properties of magneto-electro-thermo-elastic multilayer composites. Int. J. Eng. Sci..

[38] Ansari R., Gholami R. (2017). Size-Dependent Buckling and Postbuckling Analyses of First-Order Shear Deformable Magneto-Electro-Thermo Elastic Nanoplates Based on the Nonlocal Elasticity Theory. Int. J. Struct. Stab. Dyn..

[39] Ansari R., Gholami R. (2016). Nonlocal free vibration in the pre- and post-buckled states of magneto-electro-thermo elastic rectangular nanoplates with various edge conditions. Smart Mater. Struct..

[40] Lei Z., Liew K., Yu J. (2013). Buckling analysis of functionally graded carbon nanotube-reinforced composite plates using the element-free kp-Ritz method. Compos. Struct..

[41] Mota A.F., Loja M.A.R., Barbosa J.I., Rodrigues J.A. (2020). Porous Functionally Graded Plates: An Assessment of the Influence of Shear Correction Factor on Static Behavior. Math. Comput. Appl..

[42] Zhong Z., Yu T. (2006). Vibration of a simply supported functionally graded piezoelectric rectangular plate. Smart Mater. Struct..

[43] Tomczyk B., Szczerba P. (2018). Combined asymptotic-tolerance modelling of dynamic problems for functionally graded shells. Compos. Struct..

[44] Tomczyk B., Szczerba P. (2018). A new asymptotic-tolerance model of dynamic and stability problems for longitudinally graded cylindrical shells. Compos. Struct..

[45] Kondaiah P., Shankar K., Ganesan N. (2012). Pyroelectric and pyromagnetic effects on multiphase magneto–electro–elastic cylindrical shells for axisymmetric temperature. Smart Mater. Struct..

[46] Malikan M., Eremeyev V.A. (2020). On the Dynamics of a Visco–Piezo–Flexoelectric Nanobeam. Symmetry.

[47] Malikan M., Eremeyev V.A. (2020). On Nonlinear Bending Study of a Piezo-Flexomagnetic Nanobeam Based on an Analytical-Numerical Solution. Nanomaterials.

[48] Bacciocchi M., Tarantino A. (2019). Natural Frequency Analysis of Functionally Graded Orthotropic Cross-Ply Plates Based on the Finite Element Method. Math. Comput. Appl..

[49] Uzun B., Civalek O. (2019). Nonlocal FEM Formulation for Vibration Analysis of Nanowires on Elastic Matrix with Different Materials. Math. Comput. Appl..

[50] Ebrahimi F., Reza Barati M. (2016). Magnetic field effects on buckling behavior of smart size-dependent graded nanoscale beams. Eur. Phys. J. Plus.

[51] Xu X.J., Deng Z.C., Zhang K., Meng J.M. (2016). Surface effects on the bending, buckling and free vibration analysis of magneto-electro-elastic beams. Acta Mech..

[52] Heyliger P., Ramirez G. (2000). Free Vibration of Laminated Circular Piezoelectric Plates and Discs. J. Sound Vib..

[53] Mohammadimehr M., Rostami R. (2017). Bending, buckling, and forced vibration analyses of nonlocal nanocomposite microplate using TSDT considering MEE properties dependent to various volume fractions of CoFe_2_O_4_-BaTiO_3_. J. Theor. Appl. Mech..

[54] Farahmand H., Naseralavi S.S., Iranmanesh A., Mohammadi M. (2016). Navier Solution for Buckling Analysis of Size-Dependent Functionally Graded Micro-Plates. Lat. Am. J. Solids Struct..

[55] Altenbach H., Eremeyev V.A. (2011). On the shell theory on the nanoscale with surface stresses. Int. J. Eng. Sci..

[56] Ghobadi A., Golestanian H., Beni Y.T., Kamil Żur K. (2020). On the size-dependent nonlinear thermo-electro-mechanical free vibration analysis of functionally graded flexoelectric nano-plate. Commun. Nonlinear Sci. Numer. Simul..

[57] Simões Moita J.M., Mota Soares C.M., Mota Soares C.A. (2009). Analyses of magneto-electro-elastic plates using a higher order finite element model. Compos. Struct..

[58] Jędrysiak J. (2003). Free vibrations of thin periodic plates interacting with an elastic periodic foundation. Int. J. Mech. Sci..

[59] Zhou X., Yu D., Shao X., Wang S., Zhang S. (2015). Simplified-super-element-method for analyzing free flexural vibration characteristics of periodically stiffened-thin-plate filled with viscoelastic damping material. Thin-Walled Struct..

[60] Wirowski A., Michalak B., Gajdzicki M. (2015). Dynamic Modelling of Annular Plates of Functionally Graded Structure Resting on Elastic Heterogeneous Foundation with Two Modules. J. Mech..

[61] Michalak B. (2015). 2D tolerance and asymptotic models in elastodynamics of a thin-walled structure with dense system of ribs. Arch. Civ. Mech. Eng..

[62] Tomczyk B., Szczerba P. (2017). Tolerance and asymptotic modelling of dynamic problems for thin microstructured transversally graded shells. Compos. Struct..

[63] Xiang H.J., Shi Z.F. (2009). Analysis of flexural vibration band gaps in periodic beams using differential quadrature method. Comput. Struct..

[64] Xu Y., Zhou X., Wang W., Wang L., Peng F., Li B. (2016). On natural frequencies of non-uniform beams modulated by finite periodic cells. Phys. Lett. A.

[65] Jędrysiak J. (2020). Tolerance Modelling of Vibrations and Stability for Periodic Slender Visco-Elastic Beams on a Foundation with Damping. Revisiting. Materials.

[66] Jędrysiak J. (2017). Tolerance modelling of free vibration frequencies of thin functionally graded plates with one-directional microstructure. Compos. Struct..

[67] Marczak J., Jędrysiak J. (2018). Some remarks on modelling of vibrations of periodic sandwich structures with inert core. Compos. Struct..

[68] Wu Z.J., Li F.M., Wang Y.Z. (2014). Vibration band gap properties of periodic Mindlin plate structure using the spectral element method. Meccanica.

[69] Cheng Z., Xu Y., Zhang L. (2015). Analysis of flexural wave bandgaps in periodic plate structures using differential quadrature element method. Int. J. Mech. Sci..

[70] Zhou X., Yu D., Shao X., Wang S., Tian Y. (2014). Band gap characteristics of periodically stiffened-thin-plate based on center-finite-difference-method. Thin-Walled Struct..

[71] Yu D., Wen J., Shen H., Xiao Y., Wen X. (2012). Propagation of flexural wave in periodic beam on elastic foundations. Phys. Lett. A.

[72] Chen T. (2013). Investigations on flexural wave propagation of a periodic beam using multi-reflection method. Arch. Appl. Mech..

[73] Wang Q. (2002). On buckling of column structures with a pair of piezoelectric layers. Eng. Struct..

[74] Li Y. (2014). Buckling analysis of magnetoelectroelastic plate resting on Pasternak elastic foundation. Mech. Res. Commun..

[75] Park W.T., Han S.C. (2018). Buckling analysis of nano-scale magneto-electro-elastic plates using the nonlocal elasticity theory. Adv. Mech. Eng..

[76] Ramirez F., Heyliger P.R., Pan E. (2006). Discrete Layer Solution to Free Vibrations of Functionally Graded Magneto-Electro-Elastic Plates. Mech. Adv. Mater. Struct..

